# Use of COVIDTests.gov At-Home Test Kits Among Adults in a National Household Probability Sample — United States, 2022

**DOI:** 10.15585/mmwr.mm7216a6

**Published:** 2023-04-21

**Authors:** Nicole Luisi, Patrick S. Sullivan, Travis Sanchez, Heather Bradley, Mansour Fahimi, Kayoko Shioda, Kristin N. Nelson, Benjamin A. Lopman, Aaron J. Siegler

**Affiliations:** ^1^Department of Epidemiology, Rollins School of Public Health, Emory University, Atlanta, Georgia; ^2^Marketing Systems Group, Philadelphia, Pennsylvania; ^3^Gangarosa Department of Environmental Health, Rollins School of Public Health, Emory University, Atlanta, Georgia.

At-home rapid antigen COVID-19 tests were first authorized by the Food and Drug Administration in late 2020 (*1*–*3*). In January 2022, the White House launched COVIDTests.gov, which made all U.S. households eligible to receive free-to-the-user at-home test kits distributed by the U.S. Postal Service (*2*). By May 2022, more than 70 million test kit packages had been shipped to households across the United States (*2*); however, how these kits were used, and which groups were using them, has not been reported. Data from a national probability survey of U.S. households (COVIDVu), collected during April–May 2022, were used to evaluate awareness about and use of these test kits (*4*). Most respondent households (93.8%) were aware of the program, and more than one half (59.9%) had ordered kits. Among persons who received testing for COVID-19 during the preceding 6 months, 38.3% used a COVIDTests.gov kit. Among kit users, 95.5% rated the experience as acceptable, and 23.6% reported being unlikely to have tested without the COVIDTests.gov program. Use of COVIDTests.gov kits was similar among racial and ethnic groups (42.1% non-Hispanic Black or African American [Black]; 41.5% Hispanic or Latino [Hispanic]; 34.8% non-Hispanic White [White]; and 53.7% non-Hispanic other races [other races]). Use of other home COVID-19 tests differed by race and ethnicity (11.8% Black, 44.4% Hispanic, 45.8% White, 43.8% other races). Compared with White persons, Black persons were 72% less likely to use other home test kits (adjusted relative risk [aRR] = 0.28; 95% CI = 0.16–0.50). Provision of tests through this well-publicized program likely improved use of COVID-19 home testing and health equity in the United States, particularly among Black persons. National programs to address availability and accessibility of critical health services in a pandemic response have substantial health value.

Methods of the national COVIDVu survey have been previously reported (*4*). In brief, a national address-based household sample derived from the U.S. Postal Service Computerized Delivery Sequence File, which covers nearly all residential delivery points in all 50 U.S. states, was recruited. During August–December 2020, 39,500 addresses were selected in a probability sample, and one household member aged ≥18 years from each address was randomly selected to participate (*4*,*5*). Among 5,666 (15.3%) respondents who completed a baseline survey, 4,654 (12.6% of sampled households; 82.1% of those who completed a baseline survey) were eligible to participate in follow-up survey rounds, based on completion of study procedures (*5*). Weights were derived using raking* and trimming^†^ procedures. Demographic and geographic data were used to develop person- and household-level weights, with additional adjustments for kit ordering using the publicly available figure of 70 million COVIDTests.gov orders shipped to households (*2*). Extreme weights were trimmed and readjusted to reach the total sum of households and noninstitutionalized adults aged ≥18 years in the United States. Among 4,654 participants enrolled at baseline (*5*), 3,400 (73%) completed an online follow-up survey during April 14–May 13, 2022. Data from the 3,400 responses were weighted to allow for national estimates of COVIDTests.gov use among U.S. households (128,674,487) and adults aged ≥18 years (252,117,111). Participants were asked to specify any COVID-19 tests they had received during the previous 6 months (e.g., laboratory, pharmacy, doctor’s office, drive through site, government home test kit, or other home test kit). Awareness of the COVIDTests.gov program was assessed based on response to the question “Did you know that the government is offering free COVID-19 home test kits that can be ordered and mailed to your home?” Participants who answered “Yes” were asked if their household had ordered any of these government test kits, and whether these kits had been used by them or someone else. Those who used a government kit to self-administer a test during the previous 6 months were asked questions about the acceptability and use of the test kit. Sociodemographic variables were self-reported at baseline; weighted estimates and two-sided 95% CIs were prepared for the full sample and for the subset of respondents reporting use of a COVIDTests.gov kit. To evaluate the association between race and ethnicity and different testing modalities, aRRs and 95% CIs were estimated using weighted multivariable negative binomial regression; models were adjusted for age, sex, region, education, income, household size, and vaccination status.

All analyses were conducted using SAS statistical software (version 9.4; SAS Institute). The COVIDVu study was approved by the Emory University Institutional Review Board.

In May 2022, an estimated 93.8% (120,730,524) of U.S. households were aware of the COVIDTests.gov program, and 59.9% (77,089,010) of households had ordered government kits (Figure 1). In nearly one third (32.1%; 41,325,184) of households, at least one government test kit had been used by someone within or outside the home. An estimated 27.8% (35,763,825) of households had ordered COVIDTests.gov kits but had not yet used them.

**FIGURE 1:**
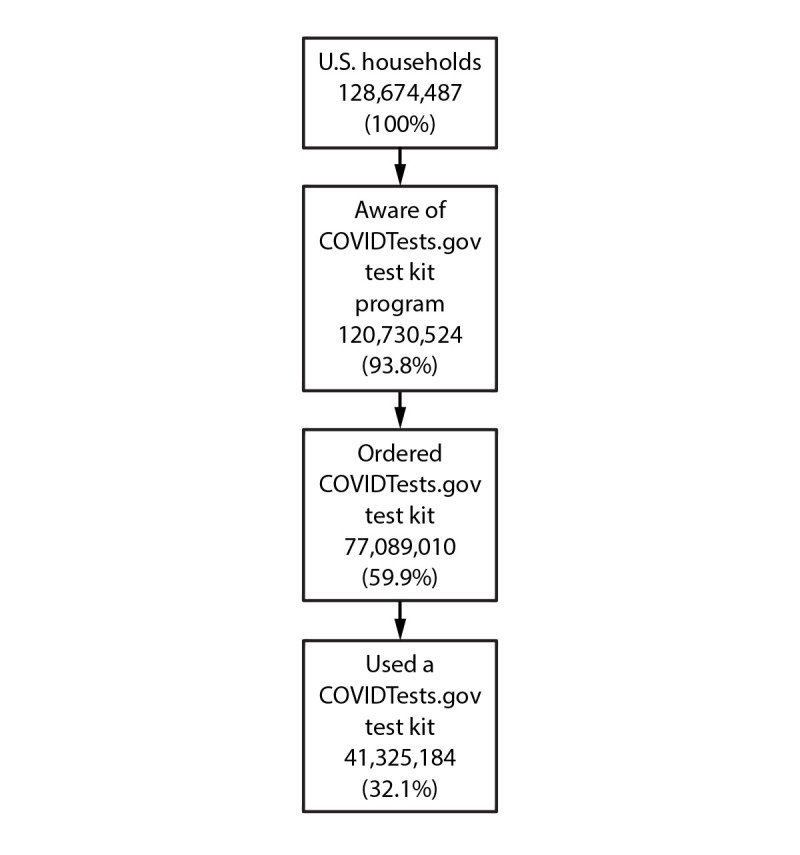
Estimated COVIDTests.gov test kit awareness, ordering, and use by household*^,^^†^^,^^§^ — United States, April–May 2022^¶^<Fig_Small></Fig_Small> * Based on a national probability sample (COVIDVu). ^†^ Household-level weights were derived with raking and a trimming procedure. Demographic and geographic data were used to develop person-level weights, which were adjusted to create household-level weights based on the joint distribution of households within each geographic region of the United States, total number of adults and children in the home, household income, and home ownership. The publicly available benchmark of 70 million household COVIDTests.gov orders was used to adjust the person- and household-level weights. Extreme weights were trimmed and readjusted to sum to 128 million total U.S. households. ^§^ Test kit use includes any COVIDTests.gov kit use by resident or nonresident of household. ^¶^ Percentage of estimated total 128,674,487 U.S. households.

Among an estimated 252,117,111 adults, 154,160,315 (61.1%) lived in a household that had ordered kits (Table), 41.3% (63,663,434) of whom had self-administered a test kit during the preceding 6 months. Approximately one half of persons aged 18–44 years had used kits (54.6% of those aged 18–34 and 50.9% of those aged 35–44 years), compared with 35.2%, 42.1%, and 26.8% of persons aged 45–54, 55–64, and ≥65 years, respectively. The lowest kit use prevalence was in the South U.S. Census Bureau Region (35.2%), and the highest was in the West U.S. Census Bureau Region (47.4%). Among households including three or more persons, approximately one half (46.0%) used kits, compared with 38.2% of households of fewer than three persons.

**TABLE:** Characteristics of adults* surveyed about SARS-CoV-2 testing and COVIDTests.gov kit use *—* United States, April–May 2022

Characteristic	All adults (N = 252,117,111)		Used a COVIDTests.gov test kit (n = 63,663,434)	
	Weighted, no.	Weighted, % (95% CI)^†^	Weighted, no.	Weighted, % (95% CI)^§^
**COVIDTests.gov kits ordered within household**	154,160,315	61.1 (58.0–64.2)	63,663,434	41.3 (38.0–44.7)
**Race and ethnicity**				
Black or African American, non-Hispanic	25,850,841	10.3 (8.4–12.5)	6,858,446	41.4 (30.2–53.6)
White, non-Hispanic	164,393,188	65.2 (62.2–68.1)	37,028,796	38.4 (34.6–42.3)
Hispanic or Latino	40,170,347	15.9 (13.8–18.4)	11,944,942	47.8 (38.9–56.8)
Other races, non-Hispanic	21,702,735	8.6 (7.0–10.5)	7,831,250	48.5 (37.4–59.8)
**Sex**				
Female	136,155,934	54.0 (51.0–57.0)	38,013,147	42.0 (37.8–46.3)
Male	115,961,177	46.0 (43.0–49.0)	25,650,287	40.3 (35.0–46.0)
**Age group, yrs**				
18–34	69,765,556	27.7 (25.0–30.6)	18,049,996	54.6 (47.1–61.9)
35–44	42,951,172	17.0 (14.9–19.4)	13,559,996	50.9 (43.0–58.7)
45–54	40,515,793	16.1 (14.1–18.3)	10,029,549	35.2 (27.9–43.3)
55–64	42,767,176	17.0 (15.0–19.2)	11,973,956	42.1 (34.7–49.9)
≥65	56,117,414	22.3 (19.9–24.8)	10,049,937	26.8 (21.3–33.0)
**U.S. Census Bureau region^¶^**				
Northeast	44,036,175	17.5 (15.2–19.9)	14,330,946	46.3 (38.3–54.5)
Midwest	53,575,092	21.3 (18.8–23.9)	11,451,840	40.0 (32.3–48.3)
South	92,920,639	36.9 (34.0–39.8)	20,006,130	35.2 (29.8–41.0)
West	61,585,205	24.4 (22.1–26.9)	17,874,519	47.4 (41.7–53.2)
**Education**				
High school diploma, GED, or less	84,356,738	33.5 (30.3–36.8)	18,849,902	39.5 (31.7–47.8)
Some college or associate degree	73,360,945	29.1 (26.6–31.7)	16,772,376	40.6 (35.0–46.4)
Bachelor's degree	59,827,173	23.7 (21.7–25.9)	17,166,926	42.9 (37.8–48.1)
Graduate degree	34,572,255	13.7 (12.3–15.3)	10,874,231	43.4 (37.9–49.1)
**Income, US$**				
0–24,999	30,092,036	11.9 (10.1–14.0)	5,463,583	41.0 (31.0–51.7)
25,000–49,999	42,687,868	16.9 (14.7–19.4)	9,413,207	39.3 (30.9–48.5)
50,000–99,999	73,698,765	29.2 (26.6–32.0)	20,287,832	44.0 (37.8–50.4)
100,000–199,999	72,574,972	28.8 (26.2–31.5)	20,189,928	42.5 (36.5–48.8)
≥200,000	33,063,470	13.1 (11.3–15.1)	8,308,883	35.6 (28.4–43.6)
**Household size**				
1–2 persons	151,075,517	59.9 (56.9–62.8)	35,303,476	38.2 (34.1–42.4)
≥3 persons	101,041,594	40.1 (37.2–43.1)	28,359,958	46.0 (40.5–51.7)
**No. of COVID-19 vaccine doses received**				
None	24,619,675	9.8 (7.9–12.0)	3,650,426	36.3 (22.5–52.6)
1	8,589,424	3.4 (2.4–4.7)	1,485,647	42.5 (26.3–60.4)
2	59,427,771	23.6 (21.0–26.4)	11,371,227	40.3 (32.1–49.0)
3	143,095,746	56.8 (53.7–59.7)	43,357,811	43.0 (39.0–47.2)
>3	16,384,496	6.5 (5.2–8.0)	3,798,322	32.7 (23.6–43.3)
**COVID-19 testing, previous 6 mos**				
Did not test	85,988,644	34.1 (31.3–37.1)	NA	NA
Tested, used at least one COVIDTests.gov test	63,663,434	25.3 (22.9–27.8)	NA	NA
Tested, did not use a COVIDTests.gov test	102,465,033	40.6 (37.7–43.6)	NA	NA
COVIDTests.gov test kit use in household **				
Used for self	63,663,434	41.3 (38.0–44.7)	NA	NA
Used by household member	42,511,539	27.6 (25.0–31.0)	NA	NA
Used by someone else	5,823,485	3.8 (2.7–5.3)	NA	NA
Test kits haven't arrived	3,420,382	2.2 (1.2–4.1)	NA	NA
No use of test kits	70,591,623	45.8 (42.4–49.2)	NA	NA
**Other characteristics of COVIDTests.gov kit users, previous 6 mos^††^**				
**Used only COVIDTests.gov kit**	18,534,473	29.7 (24.9–35.1)	NA	NA
**Unlikely to test without COVIDTests.gov kit**	13,884,660	23.6 (19.3–28.4)	NA	NA
**Rating of COVIDTests.gov kit use experience**				
Very acceptable	32,053,174	64.1 (57.9–69.9)	NA	NA
Acceptable	15,676,552	31.4 (25.9–37.4)	NA	NA
Neutral or unacceptable	2,248,190	4.5 (2.3–8.7)	NA	NA
**Had any positive COVID-19 test result**	14,133,748	22.2 (18.1–26.9)	NA	NA

**Abbreviations:** GED = general educational development certificate; NA = not applicable.

* From a national probability sample (COVIDVu).

^†^ Percentages calculated as column percents, unless otherwise specified.

^§^ Percentages calculated as row percentages. Denominator includes only respondents living in households that ordered COVIDTests.gov test kits.

^¶^ https://www2.census.gov/geo/pdfs/maps-data/maps/reference/us_regdiv.pdf

** Categories are not mutually exclusive. Denominator includes only respondents living in households that ordered COVIDTests.gov test kits.

^††^ Denominator includes only respondents who used COVIDTests.gov test kits. Denominators in this section excluded the following missing values: used only COVIDTests.gov kit, n = 1,299,627; unlikely to test without COVIDTests.gov kit, n = 4,713,097; and rating of COVIDTests.gov kit use experience, n = 13,685,518.

Nearly one quarter (23.6%) of persons using the government kits (more than 13 million persons) indicated that they would have been unlikely to test for COVID-19 if COVIDTests.gov kits were not available; 29.7% of kit users had not used any other type of test during the preceding 6 months. The survey did not collect information about test results from each test used, but 22.2% of COVIDTests.gov kit users reported at least one positive test result within this period. Among those who used a COVIDTests.gov kit, more than 95% rated the experience as very acceptable (64.1%) or acceptable (31.4%).

Among 166,128,467 (65.9%) adults who received testing for COVID-19 during the previous 6 months, 44.2% received a laboratory or clinic test, 25.7% received a drive-through test, 38.3% used a COVIDTests.gov kit, and 42.1% used another type of at-home self-test kit (e.g., pharmacy) (Figure 2). Prevalences of COVIDTests.gov kit use differed slightly among Black (42.1%), Hispanic (41.5%), White (34.8%), and other race persons (53.7%) (p = 0.03); compared with White persons, persons of other races were more likely to use government kits (aRR = 1.42; 95% CI = 1.12–1.80; p = 0.004), but Black and Hispanic persons were not. This differed from the use of other at-home self-test kits, with considerably lower use among Black (11.8%) than among Hispanic (44.4%), White (45.8%), and persons of other races (43.8%). Black persons were 72% less likely to use other home test kits compared with White persons (aRR = 0.28; 95% CI = 0.16–0.50; p<0.001), despite similar use of COVIDTests.gov kits.

**FIGURE 2:**
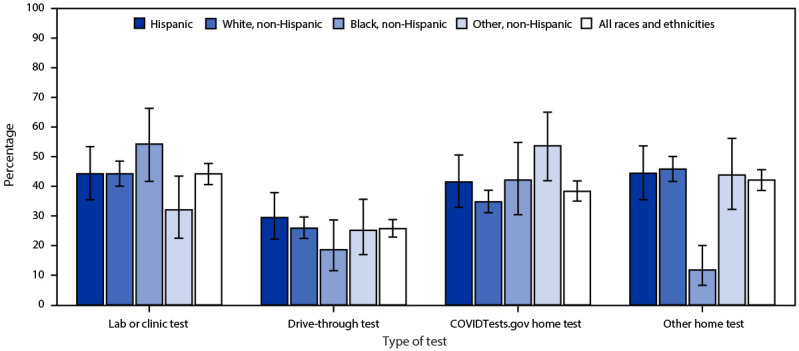
Modalities of COVID-19 testing*^,^**^†^** among adults who received testing for COVID-19 during the previous 6 months (n = 166,128,467),^§^ by race and ethnicity — COVIDVu,^¶^ United States, April–May 2022 *Lab or clinic tests were used by 72,777,029 (44.2%), drive-through tests by 42,386,207 (25.7%), COVIDTests.gov tests by 63,663,434 (38.3%) and other home tests by 69,331,543 (42.1%) persons who received testing during the previous 6 months. ^†^ With 95% CI indicated by error bars. ^§^ Data for use of lab or clinic, drive-through, and other home tests were missing for a small proportion (<1%) of respondents who received testing during the previous 6 months; the denominator for these tests is 164,828,840. ^¶^ A national probability sample.

## Discussion

In a probability sample of U.S. households, both awareness of the COVIDTests.gov program and use of kits were high, with more than 41 million households estimated to have used kits during January–May 2022. This number is likely a lower bound estimate because households might not have had sufficient time to use their ordered test kits at the time of the survey.

Prevalences of COVID-19 testing using the COVIDTests.gov kits were similar among racial and ethnic groups, a considerable difference from the highly inequitable use of other home self-tests for Black respondents observed both in this survey and in previous estimates (*3*). Racial and ethnic disparities in COVID-19 illness have been widely reported (*6*), as have concerns about the cost and accessibility of at-home tests (*7*). Long-term availability of government test kits appears to have significantly improved access to COVID-19 testing for racial and ethnic minorities, underscoring a critically important and successful element of the national COVID-19 response.

The findings in this report are subject to at least one limitation. Survey weights were used to establish estimates that represent the U.S. population; however, COVIDVu respondents might have been more likely to practice preventive measures such as vaccination and testing. To adjust for this potential bias, publicly available data on COVIDTests.gov kit orders were used as part of the weighting process, ensuring that the overall estimate of kit ordering is realistic; however, other sources of bias not addressed by weighting procedures might remain.

These data indicate that provision of free COVID-19 tests through the COVIDTests.gov program was not only widely used, but also provided a mechanism for millions of persons to receive COVID-19 testing who otherwise might not have. Moreover, this program likely led to improvement in equity of COVID-19 testing. The COVIDTests.gov program was provided continuously until September 2, 2022, when it was paused; the site was reactivated on December 19, 2022 (*8*). These findings support the substantial health value of national programs that address critical health needs during a pandemic response.

**Summary**

**What is already known about this topic?**

During January 2022, the White House launched COVIDTests.gov, a program through which all U.S. households could order free-to-the-user at-home test kits from the federal government, distributed by the U.S. Postal Service.

**What is added by this report?**

Awareness and acceptability of the COVIDTests.gov program is high. COVIDTests.gov test kits have improved access to COVID-19 testing, with more than 40 million households using at least one kit. The program helped to improve equity of COVID-19 home test use; non-Hispanic Black or African American (Black) and non-Hispanic White persons had similar use of COVIDTests.gov kits, and Black persons were 72% less likely to use other modalities for home-based testing.

**What are the implications for public health practice?**

National programs to address availability and accessibility of critical health services in a pandemic response have substantial health value.

## Footnotes

* Raking is an iterative process that assigns a weight value to each survey respondent to ensure that the weighted distribution of the sample more closely approximates that of the sampled population. This procedure was used to align distributions with respect to gender, age, race, ethnicity, education, income, marital status, and U.S. Census Bureau region.

† Trimming is a process applied during raking to control the highest and lowest weight values. At both ends of the distribution, weights were trimmed at the 99th percentile.
